# Association Between Lateralized Periodic Discharge Amplitude and Seizure on Continuous EEG Monitoring in Patients With Structural Brain Abnormality in Critical Illness

**DOI:** 10.3389/fneur.2022.840247

**Published:** 2022-03-16

**Authors:** Safoora Fatima, Mengzhen Sun, Klevest Gjini, Aaron F. Struck

**Affiliations:** ^1^Department of Neurology, Wisconsin Hospital and Clinics, University of Wisconsin, Madison, WI, United States; ^2^William S. Middleton Memorial Veterans Hospital, University of Wisconsin, Madison, WI, United States

**Keywords:** LPD, seizure, amplitude, PLEDs, lateralized periodic discharge

## Abstract

**Objective:**

To investigate the association between lateralized periodic discharge (LPD) amplitude and seizure risk on an individual level in patients with structural brain abnormality.

**Methods:**

Retrospective case–control study of patients with structural brain abnormality undergoing continuous EEG monitoring was performed. We included 10 patients with LPDs and seizures as cases and 10 controls, patients with LPDs without seizure. Analysis was performed with a mixed-effects model with primary outcome measure of number of seizures per 8-h EEG epoch with fixed effects being variables of interest and random effect being subject ID.

**Results:**

Epochs with seizures showed a higher absolute amplitude (corrected *p* = 0.04) and a higher relative amplitude (corrected *p* = 0.04) of LPDs. Additionally, the number of seizures was higher in epochs that had LPDs with plus features (uncorrected *p* = 0.002) and LPDs with higher relative amplitude (uncorrected *p* = 0.005).

**Conclusion:**

Higher LPD amplitude is associated with increased risk of seizures on an individual patient level. A decreasing amplitude is suggestive of decreasing seizure risk, and may in fact be suggestive of decreasing ictal character of LPDs.

## Highlights

- Lateralized periodic discharges (LPDs, also known as PLEDs) are a common EEG pattern in critically ill patients and associated with increased risk of seizures.
- On an individual patient level, decreasing LPD amplitude reflects decreasing seizure risk.
- Lateralized periodic discharge plus features are associated with increased risk of seizure.

## Introduction

Lateralized periodic discharges (LPDs), formerly known as periodic lateralized epileptiform discharges (PLEDs) were first described by Chatrain et al. as periodic or quasi-periodic unilateral focal spikes or sharp waves, occurring at a rate of 1–2 Hz (1). LPDs are commonly found in association with focal acute or subacute cerebral lesions like subdural hematoma, intracerebral hemorrhage, infectious etiologies, tumor, autoimmune conditions, metabolic disorders, and cerebrovascular disease (2). LPDs are a common EEG pattern in critically ill patients, with prevalence of ~6.2–8.6% (3). From LPDs' first description, there has been controversy whether these patterns are ictal or interictal in nature, or whether they should be viewed as a part of ictal–interictal continuum (4).

Lateralized periodic discharges are found to be associated with an increased risk of discrete seizures (5, 6). Certain electrographic features of LPDs portend a greater risk of seizures. These features associated with an increased risk of seizures may also suggest a more “ictal” nature of the LPDs, placing them closer to the ictal end of the ictal–interictal continuum (7, 8). This remains true for LPD frequency on at least superficial level. LPDs with a higher frequency are associated with both higher risk of discrete seizures and increased markers of metabolic distress (9, 10). Studies in the past have established direct correlation between LPD frequency and metabolic activity (11).

In this study, we primarily aimed to determine a relationship between LPD amplitude with seizure on an individual level. The study of LPD amplitude is difficult as there are other factors that contribute to amplitude other than spatial extent of the source generating the discharge and degree of synchrony. These are primarily the spatial orientation of the underlying dipole, variation in skull thickness, prevalence of skull defects, and the distance from the LPD-generating region to the skull (12). These confounding factors hamper group-wise comparisons. To overcome this barrier, we used a mixed-effect model using the individual patient as a random variable and compared 8-h epochs to see the association between LPD amplitude and number of seizures on an individual patient level. We also evaluated other electrographic features of LPDs beyond amplitude to explore their association with risk of seizure.

## Methods

### Study Design

This is a case–control study of 10 patients with LPDs and electrographic seizures compared with 10 patients with LPDs and no seizures. Groups were matched for age and patients with structural brain abnormality were selected. This study was approved by the University of Wisconsin-Madison institutional review board (IRB) and a waiver of consent was granted.

### Study Sample

We maintain a retrospective database of all patients undergoing continuous EEG (cEEG) monitoring at our institution. The inpatient monitoring is done using 10–20 standard electrode placement system. For this study, we reviewed our database for the patients who underwent cEEG monitoring between October 2018 and February 2020. Starting from October 2018, we selected the first 10 consecutive patients with structural brain abnormality who had LPDs and seizures on cEEG. After establishing the cases, the same database was reviewed to identify controls, i.e., consecutive patients with LPDs without seizures, who were monitored during the same period (*N* = 10), starting at October 2018 until 10 matched controls were found. Matching was done based on similar age range (±3 years) between the two groups and we coarsened the etiology to include only the patients with structural brain abnormality in the two groups.

### Definitions

Lateralized periodic discharges were defined according to the American Clinical Neurophysiology Society's (ACNS) standardized critical care EEG terminology as repetition of a waveform that is unilateral or bilateral but clearly and consistently with higher amplitude in one hemisphere and with relatively uniform morphology and duration, as well as with a clearly discernible inter-discharge interval between consecutive waveforms and recurrence of the waveform at nearly regular intervals (13). Electrographic seizures were defined as epileptiform discharges averaging >2.5 Hz for ≥10 s (>25 discharges in 10 s), or any pattern with definite evolution as defined above and lasting ≥10 s (13). LPD burden was defined as number of discharges in a specific period of time. Absolute amplitude was defined as typical voltage measured in standard longitudinal bipolar 10–20 recording in the channel in which the pattern is most readily appreciated measured from peak to trough and relative amplitude as typical ratio of voltage of the highest voltage component of the typical discharge to the voltage of the typical background between discharges, measured in the same channel and montage as absolute voltage (13). Relative amplitude was made into a binary variable with categories of <10 and ≥10.

### Data Acquisition

Electroencephalographic recordings of the seizure group and control group were reviewed. Total duration of EEG monitoring in the seizure group was 1,213 h and in control group was 461 h. Each raw EEG data record was notch-filtered at 60 Hz, a bandpass filter (1–70 Hz) was applied and reviewed in a longitudinal bipolar montage using CURRY 8 software (Compumedics Neuroscan, Charlotte, NC, USA). LPDs and seizures were initially marked by our investigators (S.F.) and (M.S.) and confirmed by board-certified neurophysiologist (A.S.). In both the groups, LPDs were marked manually for a period of 60 s every 10 min consistently for 1-h duration (i.e., marking was done starting at 1st, 11th, 21st, 31st, 41st, and 51st min of an hour, each for a duration of 60 s). This process was repeated every 8 h until the recording ended. Each record was abstracted into a set of variables for each of these 60-s periods based on the ACNS nomenclature “modifiers” that characterized specific electrographic features of the LPDs. These include frequency, prevalence, sharpness, absolute amplitude, relative amplitude, polarity, number of phases, and plus feature (shown in Table 1). These measures were scored semi-quantitatively for each 60-s period and for a discharge that looked prevalent in terms of morphology and amplitude in that period. A total of 692, 60-s periods constituting 11.5 h in the seizure group and in 328, 60-s periods constituting 5.5 h in the control group were scored. Seizures were marked throughout the recording for patients in seizure group and the total number of seizures in each 8-h EEG epoch was documented. Medical records of all the individuals in the study population were reviewed to collect the following variables: age, etiology, gender, history of seizure disorder and recent episode of seizure preceding monitoring.

**Table 1:** Electrographic features of LPDs that were abstracted for each epoch.

**LPD feature**	**Method of abstraction of LPD feature**
Frequency	Rate per second. Highest value of the range is recorded
Prevalence	Percent of epoch that includes the pattern Categorized as following based on that percent: Continuous ≥90%; Abundant 50-89%; Frequent 10–49%; Occasional 1–9%; Rare <1%
Sharpness	Measured as the duration of the sharpest discharge at the baseline in milliseconds (ms) specified for predominant phase. Categorized as: Spiky = duration of the component <70 ms; Sharp = duration of the component 70–200 ms; Blunt = duration of the component >200 ms
Absolute amplitude	Measured in standard longitudinal bipolar montage in the channel in which the pattern is most appreciated. It is measured from peak to trough. Units of measurement in microvolts
Relative amplitude	Measured as ratio of amplitude of the highest amplitude component to the amplitude of the typical background between the discharges, measured in the same channel and montage as Absolute Amplitude
Number of phases	Number of baseline crossings of a typical discharge counted in longitudinal bipolar montage. Categorize as 1/2/3
Polarity	Dominant phase judged in standard longitudinal bipolar montage, classified as positive or negative or unclear
Plus feature	Presence of superimposed fast activity or superimposed rhythmic or quasi-rhythmic delta activity with each discharge

*LPD, lateralized periodic discharges*.

### Data Analysis

The primary unit of analysis was an 8-h EEG epoch with the dependent variable being the number of seizures within each epoch. Independent variables included EEG features. A total of 127, 8-h epochs were available in the group of 10 patients who had seizures and LPDs. Of those epochs, 23 contained seizures and 100 contained LPDs. Of 23 epochs with seizures, 20 contained both LPDs and seizures. A total of 55, 8-h epochs were contained in the group of 10 patients with LPDs without seizures. Of those epochs, 39 contained LPDs and 0 contained seizures. Mixed-effects models were constructed only for epochs which contained LPDs. Mixed-effects models were created using R package lme4 (ver 1.1–26) with subject ID as a random variable and the variables of interest as fixed effects (14). For the first analysis, all 20 patients with a total of 139 epochs (all epochs containing LPDs) were used. For the secondary analysis, 100 epochs of only 10 patients with LPDs and seizures were examined. Statistics were performed in R (version 4.0.4; R Foundation for Statistical Computing). The statistical significance was a corrected *p* < 0.05 for primary outcome with false discovery rate correction for comparison of the absolute amplitude for the total group and absolute amplitude for seizure-only group (15). Other variables were considered exploratory and presented with uncorrected *p*-values.

## Results

Table 2 summarizes the clinical characteristics of the study population. Overall, subdural hemorrhage (SDH) (*n* = 4) and infection (*n* = 4) were the most common causes of LPDs. Overall, 7 subjects (5 in the seizure group and 2 in the non-seizure group) had undergone craniotomy either immediately before cEEG or had a history of craniotomy in the past. A total of 2 patients with SDH had cEEG done before SDH evacuation and 2 had cEEG after SDH evacuation. Approximately 80% of patients in both the groups (*N* = 8, *N* = 8) had a suspected clinical seizure preceding the monitoring as recorded from the patient chart. None of the patients had any positive clinical signs while showing LPDs on their cEEG monitoring. Approximately 20% had left temporal LPDs, 30% had left frontal LPDs, 20% had right frontal, 5% had left frontotemporal, 5% had left parasagittal, 5% had right parasagittal, 5% had right central, 5% had right frontotemporal, and 5% had left frontotemporal LPDs.

**Table 2:** Patient characteristics.

**Characteristics**	**LPD and seizure group, *N* = 10**	**LPD and no seizure group, *N* = 10**
Age in years (range)	61 (30–85)	62 (28–88)
Gender [Female *n* (%)]	3 (30)	6 (60)
**Etiologies**	
Ischemic stroke	1	2
SDH	3	1
CNS tumor	3	0
CNS infection	1	3
PRES	1	1
AVM	0	1
TBI	0	1
Encephalomalacia	0	1
Shunted hydrocephalus	1	0
Number of patients on IV sedation	7	6
Number of patients on AEDs	10	9

*LPD, lateralized periodic discharges; SDH, subdural hemorrhage; CNS, central nervous system; PRES, Posterior reversible encephalopathy syndrome; AVM, Arteriovenous malformations; TBI, Traumatic Brain Injury; IV, Intravenous; AEDs, Antiepileptic drugs*.

### Comparison of LPD Features Between Epochs Containing Seizures (*N* = 23) and Epochs Without Seizures (*N* = 116) for all 20 Patients

**Figure 1:**
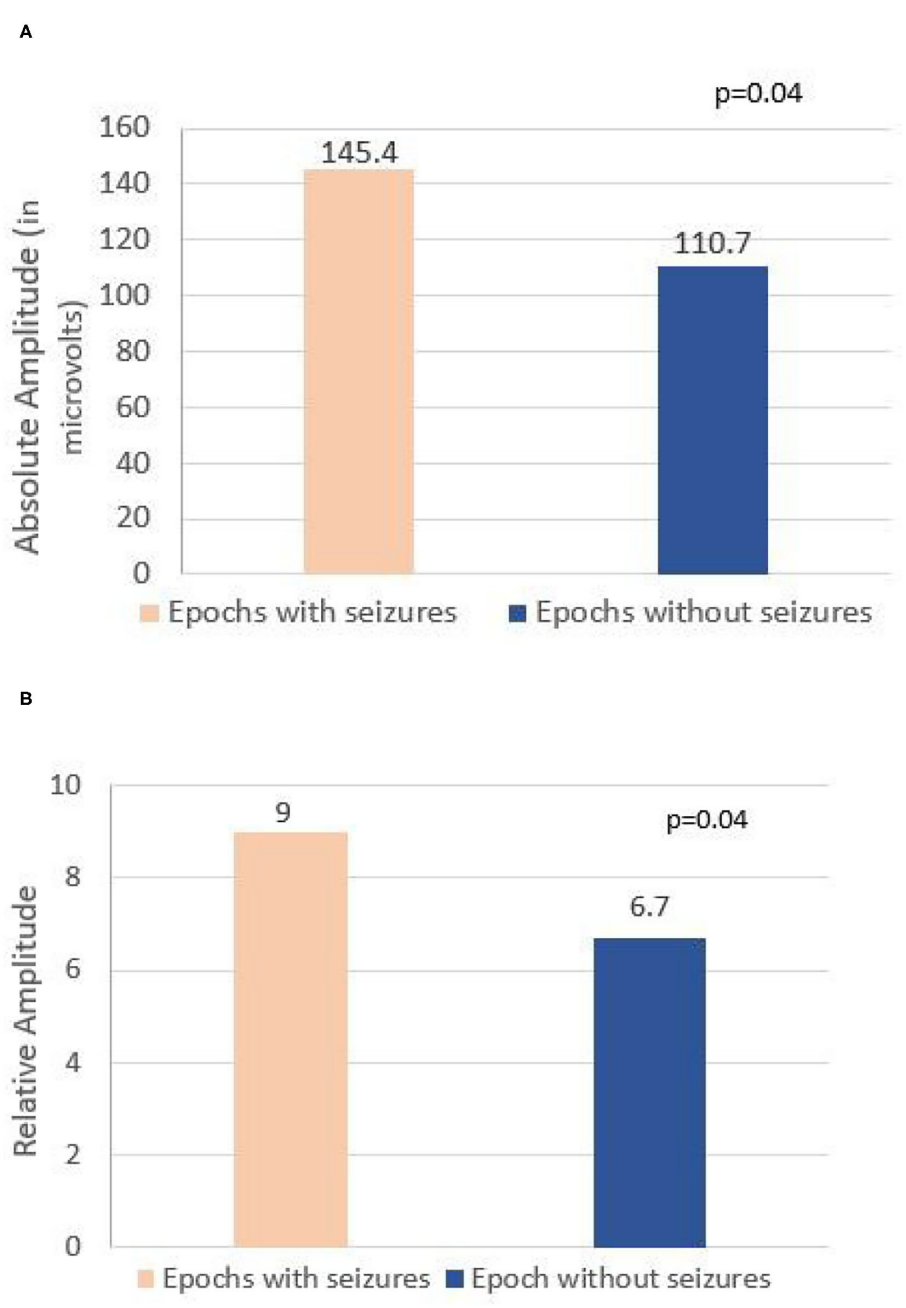
**(A)** Comparison of absolute amplitude of lateralized periodic discharges (LPDs) between epochs with seizures and epochs without seizures in all 20 patients. **(B)** Comparison of relative amplitude of LPDs between epochs with seizures and epochs without seizures in all 20 patients.

**Figure 2:**
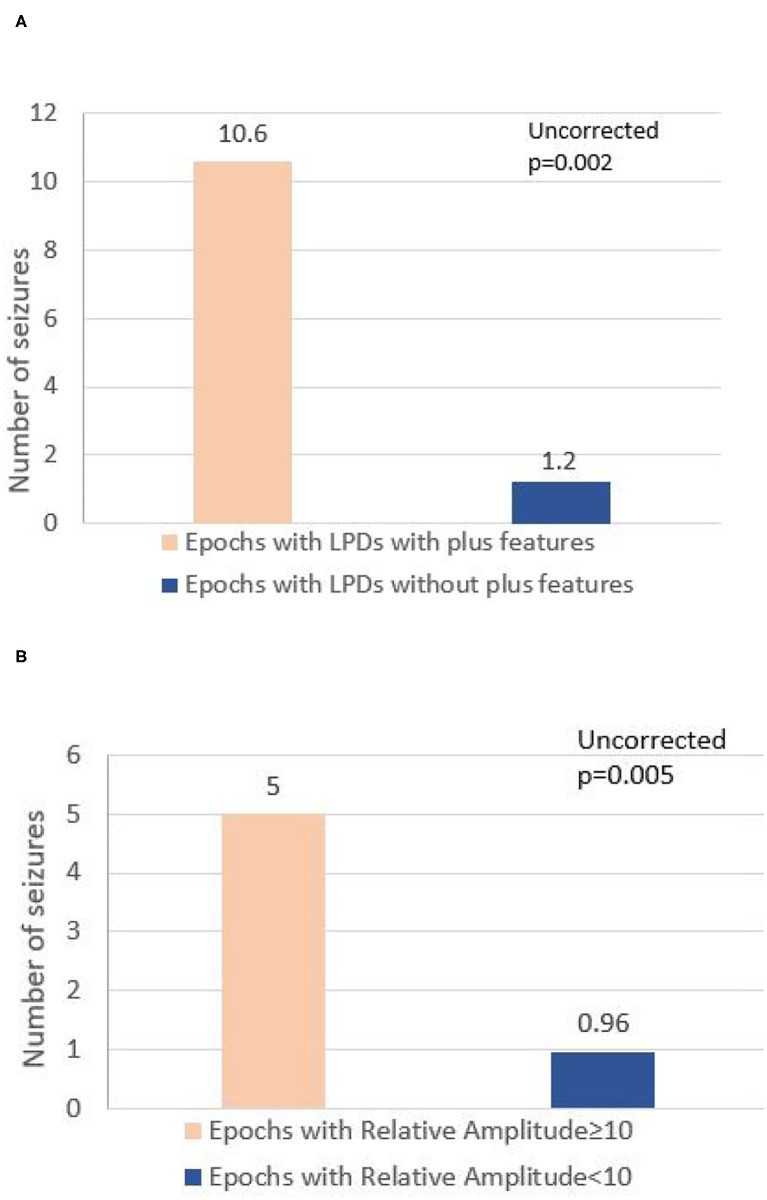
**(A)** Comparison of number of seizures between epochs with LPDs with plus features and epochs with LPDs without plus features in all 20 patients. **(B)** Comparison of number of seizures between epochs with LPD relative amplitude ≥10 and epochs with LPD of relative amplitude <10 in all 20 patients.

A total of 8-h EEG epochs from all 20 patients (10 who had seizures and 10 without seizures) were included in this analysis. Results are shown in Table 3. Absolute amplitude (*p* = 0.04 corrected) and relative amplitude (*p* = 0.04 uncorrected) of LPDs was significantly higher in the epochs that had seizures when compared to epochs without seizures, shown in Figures 1A,B. Additionally, the number of seizures was significantly higher in epochs with LPDs with the plus feature (mean 10.6) compared to those without plus feature (mean 1.2) shown in Figure 2A (uncorrected *p* = 0.002). The number of seizures was also significantly higher in epochs with LPDs of relative amplitude ≥10 (mean 5.0) compared to epochs with LPDs of relative amplitude <10 (mean 0.96) (uncorrected *p* = 0.005) shown in Figure 2B.

**Table 3:** Comparison of LPD features between epochs containing seizures (*N* = 23) and epochs without seizures (*N* = 116) for all 20 patients.

**Variable**	**Epoch with** **seizure** **Mean**	**Epoch without** **seizure** **Mean**	***P*-value**
Absolute amplitude	145.4	110.7	0.04*
Relative amplitude	9.0	6.7	0.04
LPD burden	40.0	38.0	0.88
Frequency	1.1	1.06	0.74
Prevalence			0.57
Sharpness			0.58
Polarity			0.45
Plus feature			0.002
Number of phases			0.45

*LPD, lateralized periodic discharges*.

### Comparison of LPD Features Between Epochs Containing Seizures (*N* = 23) and Epochs Without Seizures (*N* = 77) for 10 Patients With Seizures

A total of 8-h EEG epochs from only 10 patients who had seizures were included in this analysis. Results are shown in Table 4. Absolute amplitude of LPDs showed significant difference at the epoch comparison level within the seizure group (*p* = 0.04 corrected). As in the previous analysis with all patients, the number of seizures was higher in the epochs with LwPD relative amplitude ≥10 (mean 5.0) compared to relative amplitude of <10 (mean 1.5) and was again significant with an uncorrected *p* = 0.03. The Number of seizures in epochs with LPDs with the plus feature (mean 12.1) was significantly higher when compared to epochs without the plus feature (mean 1.6) with uncorrected *p* = 0.001. No significant association was found between other features of LPDs like frequency, prevalence, sharpness, burden, polarity, or number of phases with seizure risk.

**Table 4:** Comparison of LPD features between epochs containing seizures (*N* = 23) and epochs without seizures (*N* = 77) for 10 patients with seizures.

**Variable**	**Epochs with** **seizure** ** Mean**	**Epochs without** **seizures** **Mean**	***P*-value**
Absolute amplitude	145.4	119.3	0.04^*^
Relative amplitude	9.0	7.3	0.13
LPD burden	40.0	41.2	0.97
Frequency	1.10	1.10	0.84
Prevalence			0.47
Sharpness			0.80
Polarity			0.30
Plus feature			0.02
Number of phases			0.30

*LPD, Lateralized periodic discharges*;

* *means corrected for multiple comparisons*.

## Discussion

**Figure 3:**
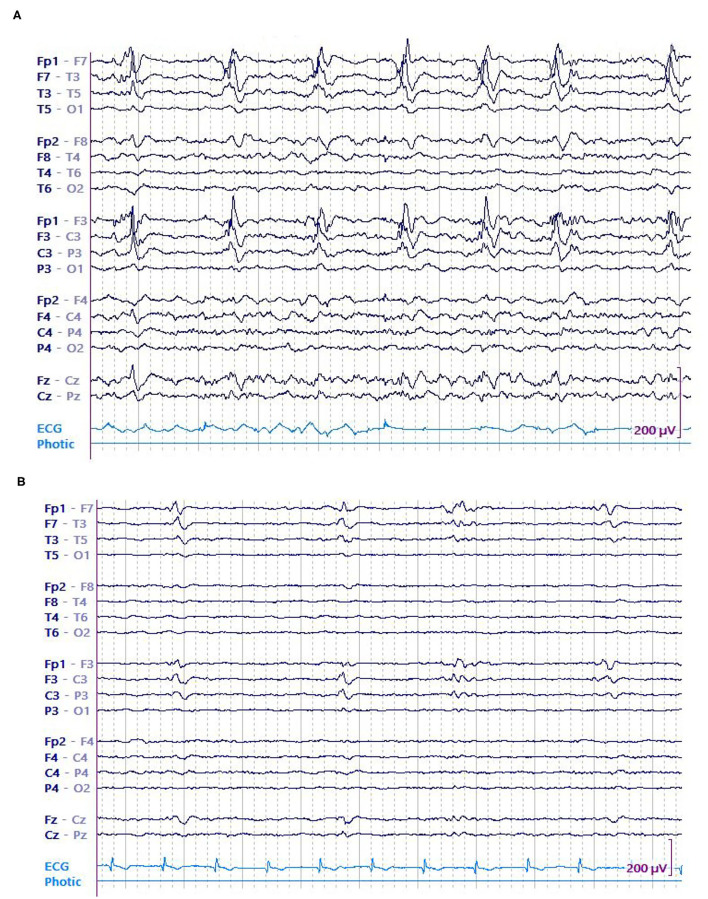
A 74 y/o gentleman with history of left frontoparietal crani for resection of the tuberculum sella meningioma a month earlier presented with dysarthria and underwent continuous EEG (cEEG) monitoring. EEG showed continuous high amplitude left frontal LPDs occurring at 0.5 Hz that evolved into recurrent left sided electrographic seizures. Patient was started on anti-seizure medications including levetiracetam, lacosamide, fosphenytoin, and clobazam. His seizures resolved eventually and LPDs decreased in amplitude concurrent with resolution of seizures. **(A)** LPDs in epoch with seizure. **(B)** LPDs in epoch without seizure.

**Figure 4:**
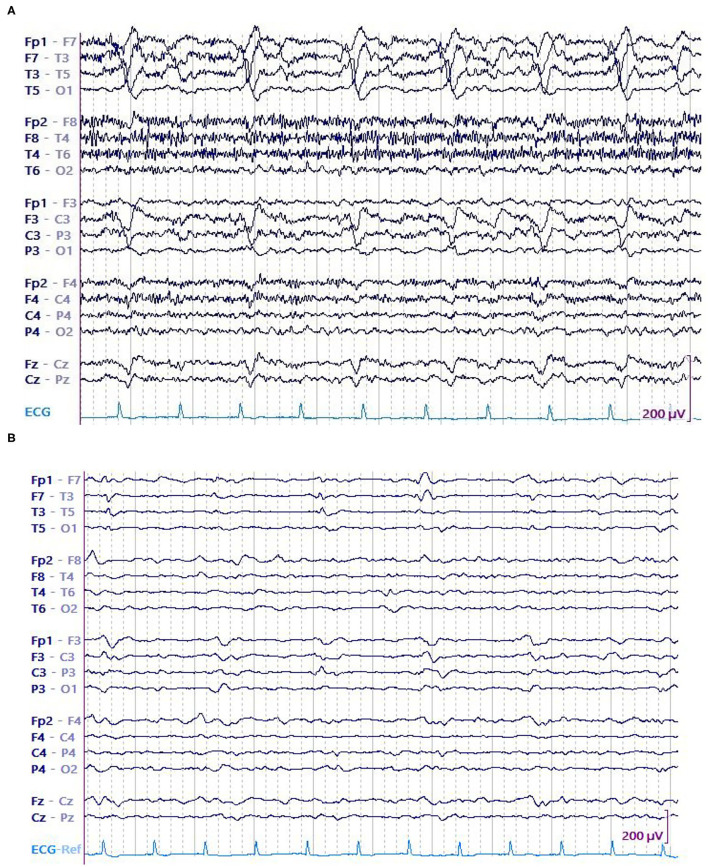
A 75 y/o woman presenting with parieto-temporal mass underwent cEEG. Her EEG showed high amplitude left temporal LPDs occurring at 0.5 Hz and brief electrographic seizures over the left temporal region. Patient was started on anti-seizure medications that include levetiracetam and valproate. After 28 h, no seizures were detected. Amplitude of the LPDs was decreased. **(A)** LPDs in epoch with seizure. **(B)** LPDs in epoch without seizure.

In this study, we primarily evaluated the association of LPD amplitude (both absolute and relative) with seizures in 8-h epochs. On an individual level, EEG epochs that had seizures were found to be associated with LPDs of higher amplitude when compared to epochs without seizures (Figures 3A,B, 4A,B). Secondarily, we evaluated the association between electrographic features of LPDs and number of seizures. We found that LPDs with higher relative amplitude and presence of plus features were associated with increased seizure numbers. Our finding suggests that LPDs with higher absolute and relative amplitude are associated with increased seizure risk. Comparing LPD amplitude between patients is confounded by several factors such as dipole orientation, skull thickness, and the distance between the LPD generating region and the scalp electrodes. As such LPD amplitude in and of itself is unlikely to contribute to improved risk stratification of methods like 2HELPS2B (10). But on an individual patient level if LPD amplitude is increasing or decreasing it suggests that the seizure risk is similarly increasing or decreasing.

The previous large studies analyzing the association of characteristics of periodic patterns with seizures did not specifically evaluate a correlation between LPD amplitude and seizure risk (10, 16). Our study differs from prior studies in methodology, in that we abstracted the LPD characteristics semi-quantitatively instead of relying on clinical reports which often do not have accurate amplitude data. To overcome the confounding factors associated with amplitude measurement, we used a mixed-effects model in which an intercept is fitted for each subject allowing for isolating the variable of interest, and found that LPD amplitude changes on an individual level are associated with seizure number. This finding suggests that LPD amplitude, which is easily quantifiable, could be tracked epoch-to-epoch on a routine clinical basis to aid the overall impression of seizure risk.

Our study also confirmed the association between LPDs with “plus” features and increased seizure risk which is well-established (10, 16–18). However, we did not find a significant relationship between other LPD features like frequency, prevalence or morphology, and seizure risk as demonstrated by previous studies (10, 16, 18, 19). This discrepancy is most likely due to the sample size and not because these are not risk factors for seizure. It is potentially interesting that these well-established seizure risk factors were not as powerful as LPD amplitude on an individual patient level in this study.

Though not directly tested in our study, it is suggested that electrographic features of LPDs like amplitude and “plus” feature, as well as other markers like frequency are not just proxies for the severity of the underlying “pro-ictal” state but may have a role in assessing the ictal nature of these discharges (8, 13). These electrographic features of LPDs can help to guide treatment. LPD amplitude should be another one of these features to follow, as decreasing amplitude is suggestive of decreased extent and/or degree of paroxysmal depolarizing shifts underlying LPD generation. Further studies to expand upon these findings would include use of multimodal monitoring to evaluate metabolic stress associated with LPD amplitude and other LPD features. Methods other than LPD amplitude like electric source imaging and slope of discharge have been proposed to quantify the scope of the depolarization block underlying the discharges in LPDs. However, electric source imaging is limited in critical care EEG as it requires expertise beyond that of a typical clinical neurophysiologist and source imaging needs patient-specific head models, electrode imaging, and increased electrode density for best accuracy. Slope of the discharge like LPD amplitude is relatively easy to quantify in routine EEG interpretation, but similar to LPD amplitude, slope is affected by dipole orientation, the distance between the LPD generating region and the scalp electrodes. Subsequent studies are needed to explore the association between LPD slope and seizure risk.

There are several limitations of this study. First, the relatively small sample size which is the reason we were unable to match one of the key variables, the gender. Second, its retrospective nature creates a probable selection bias; however, this was minimized by establishing the study size, admission criteria, and review methodology *a priori*. Third, the generalizability of this study is also limited by the fact that we selected only the patients with structural brain abnormalities. Fourth, selection of short epochs and a single discharge of LPD pattern representing the 60 s epoch of the pattern. These limitations could have contributed to our results not showing a significant association between other well-known LPD features and seizure risk (10, 16, 18, 19). Future studies replicating our findings on the large sample size are required to further our current understanding of the association between LPD amplitude and seizures. Studies using larger scale and big data approaches with automated labeling of LPDs, seizures, and LPD characteristics are needed to further explore the complex interplay between LPDs, discrete seizures, and the underlying “pro-ictal state” giving rise to the ictal–interictal continuum. Additional studies using high-density EEG arrays in patients with LPDs would allow improved localization of LPD generators for the examination of the spatial extent of LPDs in source space.

## Conclusions

In summary, this retrospective preliminary study on patients with structural brain abnormality with LPDs demonstrated an association between absolute amplitude and relative amplitude of LPD with seizure. Reduction in the absolute and relative amplitude of these discharges was associated with decreased risk of seizure on an individual level. This is one of the first studies that highlighted the direct relationship between LPD amplitude and seizure risk. In addition, other features like plus feature also show a similar association with seizures. These features can be useful to augment clinical suspicion if an LPD pattern warrants empiric treatment and may also serve as a marker of response to treatment. Future research should explore the effect of anti-seizure treatment on electrographic features of LPDs, including LPD amplitude.

## Data Availability Statement

The original contributions presented in the study are included in the article/supplementary material, further inquiries can be directed to the corresponding author.

## Ethics Statement

Ethical review and approval was not required for the study on human participants in accordance with the local legislation and institutional requirements. Written informed consent for participation was not required for this study in accordance with the national legislation and the institutional requirements.

## Author Contributions

SF: substantially contributed to design, acquisition, analysis, and drafting the manuscript. MS: contributed to study design, data acquisition, and critically revised the manuscript. KG: contributed to conception, interpretation of data, and critically revised the manuscript. AS: contributed to design, data analysis, and critically revised the manuscript for important intellectual content. All authors contributed to the article and approved the submitted version.

## Funding

This work was supported by NIH NINDS R01NS111022.

## Conflict of Interest

The authors declare that the research was conducted in the absence of any commercial or financial relationships that could be construed as a potential conflict of interest.

## Publisher's Note

All claims expressed in this article are solely those of the authors and do not necessarily represent those of their affiliated organizations, or those of the publisher, the editors and the reviewers. Any product that may be evaluated in this article, or claim that may be made by its manufacturer, is not guaranteed or endorsed by the publisher.

## Abbreviations

ACNS: American Clinical Neurophysiology Society
AEDs: Antiepileptic drugs
AVM: Arteriovenous malformations
cEEG: continuous EEG
CNS: central nervous system
IV: Intravenous
LPD: lateralized periodic discharge
PRES: Posterior reversible encephalopathy syndrome
SDH: subdural hemorrhage
TBI: Traumatic Brain Injury
h: hour
s: second.
